# Effects of tilt on cerebral hemodynamics measured by NeoDoppler in healthy neonates

**DOI:** 10.1038/s41390-020-01354-w

**Published:** 2021-01-27

**Authors:** Anders Hagen Jarmund, Siv Steinsmo Ødegård, Hans Torp, Siri Ann Nyrnes

**Affiliations:** 1 Department of Circulation and Medical Imaging (ISB), Norwegian University of Science and Technology, Trondheim, Norway; 2 Children’s Clinic, St. Olavs Hospital, Trondheim University Hospital, Trondheim, Norway

## Abstract

**Background:**

Today, there are conflicting descriptions of how neonates respond to tilt. Examining physiologic responses of cerebral blood flow velocities (BFVs) in challenging situations like a tilt requires equipment that can cope with positional changes. We aimed to characterize how healthy term neonates respond to mild cerebral hemodynamic stress induced by a 90° tilt test using the recently developed NeoDoppler ultrasound system.

**Methods:**

A small ultrasound probe was fixated to the neonatal fontanel by a cap, and measured cerebral BFV in healthy neonates during and after a 90° head-up tilt test, five min in total, at their first and second day of life. Unsupervised *k*-means cluster analysis was used to characterize common responses.

**Results:**

Fifty-six ultrasound recordings from 36 healthy term neonates were analyzed. We identified five distinct, immediate responses that were related to specific outcomes in BFV, heart rate, and pulsatility index the next two min. Among 20 neonates with two recordings, 13 presented with different responses in the two tests.

**Conclusions:**

Instant changes in cerebral BFV were detected during the head-up tilt tests, and the cluster analysis identified five different hemodynamic responses. Continuous recordings revealed that the differences between groups persisted two min after tilt.

**Impact:**

- NeoDoppler is a pulsed-wave Doppler ultrasound system with a probe fixated to the neonatal fontanel by a cap that can measure continuous cerebral blood flow velocity.
- Healthy neonates present with a range of normal immediate cerebral hemodynamic responses to a 90° head-up tilt, categorized in five groups by cluster analysis.
- This paper adds new knowledge about connection between immediate responses and prolonged responses to tilt.
- We demonstrate that the NeoDoppler ultrasound system can detect minute changes in cerebral blood flow velocity during a 90° head-up tilt.

## Introduction

Neonates are subject to tremendous stress during birth, and the subsequent transition to extrauterine life involves extensive changes in hemodynamics.^1^ Assessing the state of cerebral autoregulation in neonates is of great clinical interest as neonates vary in maturation and capacity.^2^ Better understanding of normal responses to circulatory stress in healthy neonates is needed and may provide new means for early identification of vulnerable newborns and detection of altered blood flow that may require medical interventions.^3^

In a 90° head-up tilt test, the participant is switched from horizontal to upright position. Today, there are conflicting descriptions of how neonates respond to tilt.^4^ The majority of previous studies have focused on systemic parameters only, such as heart rate (HR) and blood pressure (BP).^5–18^ Furthermore, most studies have been using small tilt angles (≤45°^5,7–13,15,17,18^) that are not representative for the stress neonates normally experience, i.e., when being lifted by their parents. Increased HR and decreased BP have been found in healthy neonates following tilt the first two weeks of life.^5,8,9,11,14–17^ Others found no change or a highly variable change in HR^7,10,12,13,18^ or slightly increased BP.^6,11^

Few studies have specifically investigated the changes in cerebral blood flow velocity (BFV) in response to tilt. Anthony et al.^19^ identified four different responses in systolic BFV after a 20° head-up or -down tilt in ten healthy neonates and 50 infants in intensive care. Also, a 40° tilt test was previously briefly described by Ipsiroglu et al.^20^ Based on our literature search (Google Scholar and articles citing Anthony et al.^19^), results have neither been reported for larger tilt angles, nor how the specific initial responses to positional change are associated to the subsequent development in BFV and related parameters for term neonates. Although several authors have measured changes in oxygenation by using spectroscopy (e.g. refs. ^21–24^), this only provides an indirect measure of cerebral blood flow and may be too inaccurate for the tilt test.^25–27^ To our knowledge, none of these studies have targeted term neonates in their first week of life, except Tran et al.^24^ who found no significant change. Studies describing how the changes in HR and BP are related to actual brain perfusion are lacking.

The fontanel of the neonate provides a unique acoustic window to the brain and can be utilized to assess cerebral structures and circulation. However, in challenging situations like a tilt test, a stable Doppler-signal acquisition may be difficult using a traditional handheld ultrasound probe. A new ultrasound system named “NeoDoppler” has recently been developed for continuous monitoring of cerebral BFV in neonates.^28^ The system can record longitudinal BFV in several depths using wide, unfocused, pulsed-wave Doppler ultrasound with a small probe fixed to the fontanel by a cap.

The aims of this study were to assess whether continuous monitoring of cerebral blood flow during and after a 90° head-up tilt test was feasible with the novel NeoDoppler ultrasound system and to characterize how the cerebral blood flow in healthy neonates is in the short (within 35 s) and “long” term (40–240 s).

## Materials and methods

### Participants and recordings

The trial was carried out at The Norwegian University of Science and Technology (NTNU) and St. Olavs hospital, University Hospital in Trondheim, Norway, with participant recruitment from 15 January to 28 August 2019. Parents with neonates born at term and staying at the maternity ward at St. Olavs hospital were invited to allow their child to participate, and 44 neonates were included. Information regarding gestational age, sex, birth weight, birth length, delivery method, APGAR scores at 1, 5, and 10 min was obtained from medical records. Neonates that developed any signs of jaundice or illness during the stay were excluded from the final analysis. Postnatal age in hours, saturation, BP, HR, and distance from the heart to the anterior fontanel in cm were measured at inclusion. Saturation, BP, and HR were measured using an Intellivue MP40 patient monitor (Philips, Germany). The saturation was measured from either foot. The non-invasive blood pressure was measured using the oscillometric technique determining the systolic, diastolic, and mean arterial pressure. Appropriately sized cuffs were chosen, i.e., the smallest cuff size covering at least 2/3 of the right upper arm.

Assessment of the quality of the ultrasound recordings was done prior to any statistical analysis and consisted of visual inspection in combination with a quality score calculated in MATLAB (MathWorks© R2017a) from the correlation coefficient between the current and the previous heartbeat, graded from 0 to 100%.^28^ Recordings with quality score <80% during the test, movement of the ultrasound probe, or disconnections during the test were defined as low-quality recordings. Sounds from the neonate introduce artefacts in the Doppler spectrogram, making automatic tracing of the velocity curve difficult, and would also lower the quality score.

### Ethics

The Regional Committee for Medical and Health Research Ethics, REC Central (Reference 2017/314), The Norwegian Directorate of Health (Reference 17/15181-11), and The Norwegian Medicines Agency (Reference 19/05458) approved the study. Written informed consent was obtained from the parents.

### The NeoDoppler research system

NeoDoppler is a new ultrasound system developed by the ultrasound group at NTNU.^28^ The research set-up consists of a customized probe, a scanner (Manus EIM-A, Aurotech Ultrasound AS, Tydal, Norway), and a user interface with display. The ultrasound probe consists of a single element transducer that operates at the frequency of 7.8 MHz and uses plane waves. It is a multigated Doppler, which makes it possible to measure bloodstream velocity in several depths simultaneously down to approximately 35 mm. The NeoDoppler ultrasound system has previously been validated against conventional ultrasound.^28^ It has been found safe to use,^28^ and well within the recommended limits for thermal and mechanical indices.^29^

If the wide sound beam at a specific depth picks up several arteries, the maximum velocity trace will represent the vessel with the highest velocity. Signals from veins and arteries can be distinguished from each other by the Doppler spectrum. No angle correction is performed. The sample volume indicates the depth where the pulsed-wave Doppler curves are obtained. Pulsatility index (PI), resistance index (RI), HR, systolic BFV, mean BFV, and end-diastolic BFV are extracted from the Doppler curves. Data processing was performed with an in-house software developed in MATLAB (MathWorks© R2017a).

**Fig. 1:**
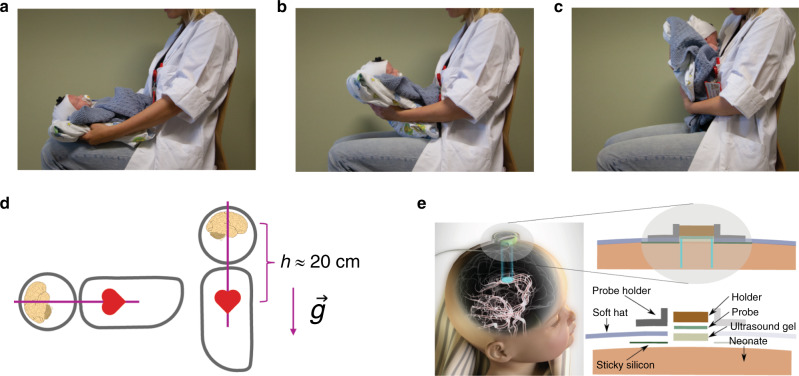
Impact of tilt and the NeoDoppler probe. **a**–**c** The 90° tilt test was performed by lifting the neonate from the supine position and up towards the chest of the tester. **d** Illustration of the impact of a head-up tilt. In the supine position, the heart and brain are in the same height. After a head-up tilt, a height difference (*h*) is introduced between the heart and brain resulting in a hydrostatic pressure because of the gravitational force $\left( {\vec g} \right)$. The corresponding hydrostatic pressure can be calculated from the heart–brain distance, gravitational constant, and the specific weight of whole blood and is approximately 15 mmHg in this study.^49^ **e** Attachment of the probe to the neonate. Each component is shown in the illustration to the right. The soft hat and the probe holder can be seen in **a**–**c**. The image to the left is by Inventas (Trondheim, Norway).

The probe housing was developed in cooperation with a product design company (Inventas, Trondheim, Norway) as described previously.^28^ The soft hat, fixating the probe, consists of Tubifast two-way stretch and parts of the inside are covered with sticky silicon (SILBIONE RT GEL 4317). The configuration is illustrated in Fig. 1. In the present study, the soft hat had more sticky silicon on the inside than in the previous study,^28^ to keep the hat better in place during the tilt test.

### Experimental protocol

**Fig. 2:**
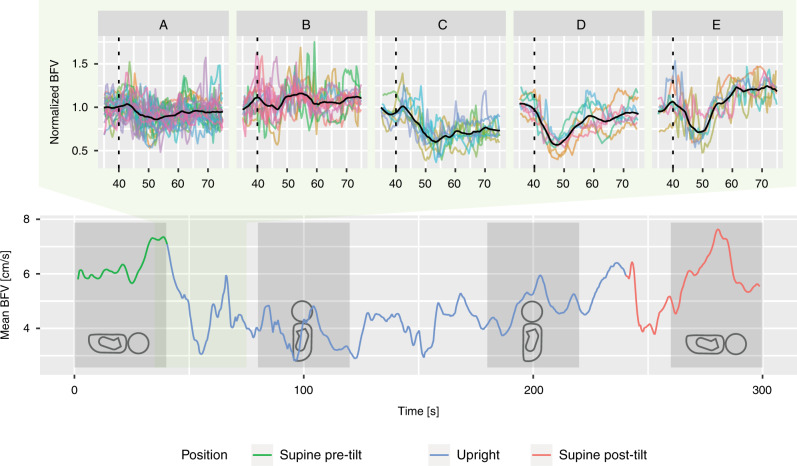
Ultrasound recording with time windows. Upper panel: Unsupervised cluster analysis revealed five distinct, initial responses to tilt (**A**–**E**). The dotted lines indicate the tilt. The tilt was performed within 2 s. Colored lines are individual recordings and the black lines are cluster means. Lower panel: A typical recording of blood flow velocity (BFV) per heartbeat during the test. The four gray boxes show the time segments (S_1_–S_4_) used for further analysis, and the green box the time window used for cluster analysis. The neonate’s position is illustrated by the silhouettes.

A tilt test was performed on newborns on two consecutive days by a doctor and a nurse in the research group. All tilts were performed as shown in Fig. 1, and the same physician (S.S.Ø.) was present during all tests to ensure consistency. The time course of the test is summarized in Fig. 2. A NeoDoppler transducer was attached to the anterior fontanel of the neonate, and the neonate was observed for 40 s before it was tilted 90° (*t* = 40 s) and kept in the upright position into the chest of the tester. After another 200 s, the child was laid down (*t* = 240 s) and recorded for additionally 60 s (*t* = 300 s). BP was measured immediately before the experiment and 80 s after the tilt (*t* = 120 s). Post-tilt BP was missing for one of the included tests.

Body surface area was calculated according to Haycock et al.^30^

### Statistical analysis

Velocity recordings were extracted from each test as elaborated above. Four time segments of 40 s were chosen for further analysis: S_1_ from 0 to 40 s, S_2_ from 80 to 120 s, S_3_ from 180 to 220 s, and S_4_ from 260 to 300 s (Fig. 2). S_1_ was regarded as baseline. PI was chosen instead of RI as it has been argued that PI is preferable in neonates.^31^ PI was calculated per heartbeat as

$${\mathrm{{PI}}} = \frac{{V_{\mathrm{{systolic}}} - V_{\mathrm{{diastolic}}}}}{{V_{\mathrm{{mean}}}}}.$$

*k*-means clustering is a much used unsupervised classification algorithm for partitioning observations into *k* groups, where the number of groups, *k*, is not known beforehand.^32^ The method is implemented for longitudinal data in the *kml* (*k*-means clustering for longitudinal data) package for R.^33,34^ Only the immediate response to tilting was of interest for making response clusters, so the cluster analysis was limited to a 40 s time window (from *t* = 35 s to *t* = 75 s) in the recordings. The recordings were normalized to baseline (S_1_) prior to cluster analysis. As the algorithm involves a random starting point that may affect the final result, 2000 redraws were used to ensure optimal cluster stability.

The optimal number of clusters was determined by running the algorithm for *k* between 2 and 10 and visually comparing quality scores provided by the *kml* package for each *k*. Five different criteria are provided by the package, and the *k* maximizing the highest number of criteria was chosen. If this resulted in clusters consisting of a single recording, the recording was regarded as an outlier and excluded, and the analysis was repeated for the remaining recordings.

Linear mixed models (LMMs) were used to take the correlation between recordings of the same neonate and between time segments extracted from the same recording into account. With LMMs, each individual and recording can have its own intercept and slope modeled as a random effect. Here, participant and day were used as random intercepts. Analyses were carried out using the *lme4* package for R.^35^ Confidence intervals were acquired using bootstrapping (*n* = 1000) with the *bootpredictlme4* package.^36^ Normality of the residuals was assessed by visual inspection of QQ plots. Mean BFV and HR had to be log-transformed for their residuals to be approximately normally distributed. When population means were estimated, the average depth of all recordings was used to adjust for the effect of depth on BFV and PI. Change in baseline HR and BP was assessed with paired Student’s *t*-test with recordings included for both days and visual inspection of QQ plots to ensure normality.

## Results

### Participants and recordings

Successful monitoring during tilt was obtained in 36 of 44 neonates (82%) and 56 recordings were analyzed (Supplemental Fig. S1). Twenty neonates had recordings included from both days. Twenty-four recordings were excluded due to a calculated quality score below 80% as described above. The reasons of a low-quality score were excessive movement of the child, sounds or crying from the child, or a small displacement of the probe during the tilt test. Five exclusions were caused by computer overload during recording. In addition, five recordings were excluded because of a medical condition diagnosed after inclusion, and one recording because of deviation from the protocol. Two recordings were identified as outliers and excluded during cluster analysis as described below.

Clinical data at the time of inclusion are summarized in Table 1. Mean age at the time of testing was 25 ± 7 and 48 ± 6 h on days 1 and 2, respectively. APGAR scores ranged from 5 to 10 (mean 8.8) 1 min after birth, from 8 to 10 (mean 9.7) after 5 min, and from 9 to 10 (mean 10.0) after 10 min.

**Table 1:** Clinical data for the participants (*n* = 36) at inclusion reported as mean ± standard deviation or *n* (%).

Basic clinical parameter (unit)	
Gestational age at birth (weeks)	40.4 ± 1.3
Birth weight (g)	3598 ± 435
Birth length (cm)	49.8 ± 1.9
BSA (m^2^)	0.227 ± 0.017
Head circumference (cm)	35.6 ± 1.3
Distance heart–fontanel (cm)	19.7 ± 1.4
Female (*n*)	15 (42%)
Systolic blood pressure (mmHg)	72.3 ± 8.9
Mean arterial pressure (MAP, mmHg)	52.0 ± 7.7
Diastolic blood pressure (mmHg)	40.9 ± 9.5
Heart rate (beats per min)	120.9 ± 13.4
Birth (*n*)	
Vaginal	20 (56%)
Vaginal breech	2 (6%)
Vacuum assisted	5 (14%)
Cesarean section	9 (25%)

A typical recording of mean BFV per heartbeat is shown in the lower panel of Fig. 2. Mean ± SD depth of the recorded vessels was 21.3 ± 5.7 mm. Time segment S_1_ was regarded as baseline for the neonates as the tilt had not yet been performed. It was found that mean and systolic BFV and PI increased with increasing depth of the vessel recorded. Therefore, depth was added as a fixed effect for mean and systolic BFV and PI.

### Response in hemodynamic parameters

Median (first quantile–third quantile) of mean BFV on day 1 was 5.9 (4.5–7.0) and 9.1 (6.4–10.4) cm/s for systolic BFV, and 6.9 (5.5–8.2) and 10.2 (8.1–12.2) cm/s for mean and systolic BFV, respectively, on day 2.

Five distinct patterns of change in mean cerebral BFV were identified (upper panel in Fig. 2) based on the quality scores described above (Supplementary Fig. S2). Twenty-four recordings (43%) were assigned to cluster A, 11 (20%) to cluster B, 8 (14%) to cluster C, 7 (13%) to cluster D, and 6 (11%) to cluster E. Seven of the 20 neonates with recordings from two days presented with the same response on both days. Identical clusters were found using systolic BFV instead of mean BFV, and when applying a five-beat moving average on mean BFV prior to analysis.

**Fig. 3:**
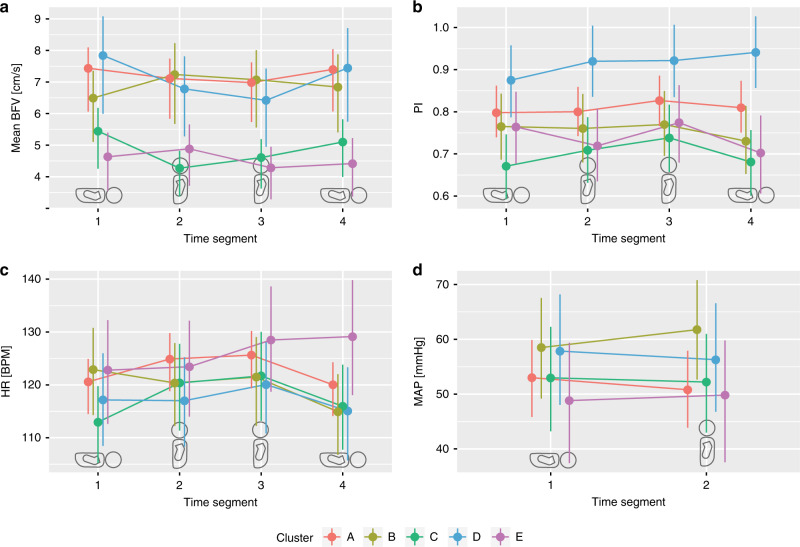
Absolute parameter estimates during the test. Linear mixed models were used to estimate mean blood flow velocity (BFV, **a**), pulsatility index (PI, **b**), heart rate (HR, **c**) in beats per min (BPM), and mean arterial pressure (MAP, **d**) at group level during four different time segments of the test (Fig. 2). The position of the neonate at each time segment is indicated by the silhouettes.

**Fig. 4:**
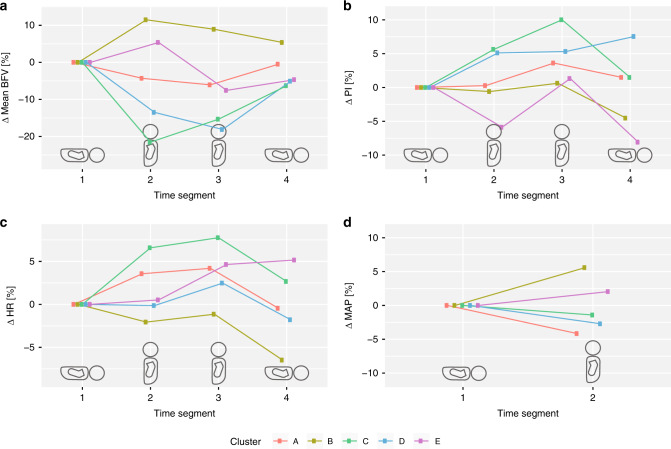
Parameter estimates during the test relative to baseline. Linear mixed models were used to estimate mean blood flow velocity (BFV, **a**), pulsatility index (PI, **b**), heart rate (HR, **c**), and mean arterial pressure (MAP, **d**) at group level during four different time segments of the test (Fig. 2). Here, the change in each parameter is shown relative to the baseline value prior to tilt (time segment S_1_). The position of the neonate at each time segment is indicated by the silhouettes.

Figures 3a and 4a show how the mean BFV in each cluster changes between the four time segments (lower panel in Fig. 2) in terms of absolute (Fig. 3a) and relative (Fig. 4a) values. Neonates in cluster C and E seem to have lower baseline BFV compared to the others. Whereas two clusters show an initial decrease in BFV of more than 10% when tilted (C and D), cluster A shows a smaller reduction of only 5%. The remaining clusters (B and E) have an initial increase in BFV but diverged as the neonate was kept upright for prolonged time. All clusters approached baseline BFV when the neonate was laid back into the supine position.

Mean PI was 0.77 ± 0.17 at day 1 and 0.79 ± 0.16 at day 2. Figures 3b and 4b show mean PI for each time segment. Cluster C and E appeared to have respectively lower and higher baseline PI than the others.

Baseline HR was 117.9 ± 11.8 and 121.5 ± 13.0 beats per min on days 1 and 2, respectively. The 20 neonates participating on both days showed a significant increase in baseline HR from day 1 to day 2 (*p* = 0.04). The development of mean HR is shown in Figs. 3c and 4c. Only cluster C had an estimated change of mean HR of more than 5% following tilt, with elevated mean HR when the neonate was kept in the upright position.

Mean arterial pressure (MAP), systolic and diastolic BP at baseline were 51.1 ± 9.3, 72.7 ± 10.3, and 38.4 ± 9.7 mmHg, respectively, on day 1 and 57.0 ± 13.8, 75.3 ± 12.3, and 46.9 ± 15.3 mmHg, respectively, on day 2. The increase in MAP from day 1 to day 2 was not significant for the 20 neonates participating on both days (*p* = 0.10). Figures 3d and 4d show estimated mean MAP before and after tilt. At group level, only cluster B had a change of mean MAP of more than 5% of baseline.

## Discussion

In this study, the NeoDoppler ultrasound system was used to record instant and prolonged changes in BFV and related parameters to 90° tilt in healthy neonates born at term. We were able to obtain at least one high-quality recording for 82% of the participants. Longitudinal cluster analysis of the BFV recordings revealed five distinct responses, and we found that the initial response was associated with differences in the subsequent development of BFV, HR, and PI. Most neonates presented with different responses on the first and second days after birth, without any apparent trends.

We used a larger tilt angle in this study than reported by previous researchers and the test procedure is more similar to what neonates usually experience (i.e. their parents lifting them up or in kangaroo care). Furthermore, this study is the first to describe how the changes in HR and BP are related to actual brain perfusion. We applied an unsupervised clustering method instead of relying on manual signal classification that was performed in former studies.^19^ While the neonatal tilt response in HR, HR variability, and indirect cerebral blood flow as assessed by spectroscopy has been investigated by many,^5–18,21–24^ only two old studies have used ultrasound to monitor brain perfusion during tilt in term neonates.^19,20^ We also found that most neonates presented with different responses on the first and second days after birth, without any apparent trends or relation to BP, as have been reported by others.^19^

The lack of change in BFV, HR, PI, and MAP for cluster A may suggest that these neonates can maintain adequate cerebral BFV, likely because of a well-functioning cerebral autoregulation.

Cluster B and cluster E differed both in terms of change of PI and BFV at baseline. It is possible that the lower baseline BFV rendered neonates in cluster E more vulnerable to hemodynamic stress, which may be reflected by the decreased PI. A lack of change in HR may suggest a weaker baroreceptor reflex. Decreased HR, as in cluster B, has been associated with a change in respiration rate.^9^

Cluster C was the only cluster displaying increase in both PI and HR shortly after tilt. Traditionally, it has been thought that the regulation of cardiac output in the neonate was only accomplished by regulating HR, as neonates have a limited capacity for adjusting their stroke volume. However, newer studies have nuanced this view.^37,38^ Thus, it is possible that the difference between clusters C and D is due to a greater ability to adjust stroke volume in cluster D, otherwise the lack of change in HR may be associated with a weaker baroreceptor reflex. The baseline BFV and PI was higher in cluster D than in cluster C. This may suggest that the neonates in cluster D tolerated a larger drop in BFV before compensatory mechanisms were evoked. A recent systematic review found no clear evidence for using single Doppler-derived parameters to predict neurological outcome in premature infants, although some evidence relates elevated RI in cerebral arteries to a hemodynamically significant ductus arteriosus (DA).^39^ We did not assess the state of the DA at the time of testing. Spontaneous closure of DA is associated with decreased PI and increased systolic and diastolic BFV in both the anterior and middle cerebral artery.^40^

The diversity of responses in this study is reflected in the literature. Anthony et al.^19^ found four distinct responses in BFV, Andrásyová et al.^41^ found four types of change in systolic BP following a 90° head-up tilt, and five response patterns have been suggested for the change in HR.^7,13^ Others have reported more consistent responses to tilt both shortly after birth and in older infants, and it is likely that the tilt response advances with postnatal age.^5,8,15,16,20,21,23,42,43^ Thus, it seems that the tilt response is not an inherent feature of the specific neonate, but arise as an interaction between the specific neonate (maturation, state of the ductal shunts, predispositions, etc.) and the specific situation (sleep/wake state, time since feeding, pressure against abdomen, hydration status, etc.). To use ultrasound in a future bedside monitoring setting, it is important to recognize the variety of normal hemodynamic responses and separate these from a pathologic response due to illness.

We were able to find two earlier reports of normal patterns of change in BFV in term neonates during tilt assessed by ultrasound. The responses described by Anthony et al.^19^ share many similarities to the five clusters identified by us. None of the neonates were totally unaffected by the tilt in this study. This may be due to a tilting angle larger than the one used by Anthony et al.,^19^ and it is possible that neonates in cluster A would be unaffected by a smaller change in posture. Also, they looked at a 20–25 s time window after the tilt, compared to 35 s in this study.

The other study, by Ipsiroglu et al.,^20^ briefly describes two distinct responses, with restoration of BFV to baseline in neonates with postnatal age greater than 4–6 weeks, but the lack of provided details makes it difficult to interpret their results.

The baseline values for BFV measured in this study are in line with previously reported measurements provided by the NeoDoppler ultrasound system.^28^ The system seems to yield slightly lower values for PI and BFV than conventional Doppler ultrasound.^31^ Some of the discrepancy may be due to lack of angle correction and basing our choice of vessels to monitor on an M-mode display only. Due to the latter, different vessels may have been insonated in the different neonates and the exact identity of the monitored artery are not known. Vik et al.^28^ investigated velocity traces extracted from several depths of the same recording and found similar Doppler velocity waveforms across depths. We believe that vessels in approximately the same anatomical location with a strong Doppler signal are representative for general brain perfusion in healthy neonates. Longitudinal monitoring focuses on intraindividual changes and less on comparing recordings between neonates. Thus, it is not necessary to insonate the same vessel in all the neonates. However, additional knowledge can be gained in future studies by comparing different monitoring methods such as Doppler ultrasound and near-infrared spectroscopy.

In this study, we assessed how the NeoDoppler ultrasound system coped with positional changes of the monitored neonate. A tilt table would provide a more standardized test setting with better control of factors such as timing, tilt velocity, and how external pressure is distributed across the neonate’s body. Manual tilting was used in this study as it is closer to how a neonate is usually managed. In our tilt tests we chose to exclude all test recordings with reduced Doppler data quality. We chose to exclude entire recordings to avoid interference with the statistical analysis, although the recordings contained parts with high quality. Dislocation of the probe as the neonate was moved, excessive movements of the neonate or sounds/crying from the neonate explained most of the exclusions. By excluding suboptimal data, we diminished the risk of that the variability in the results we observed was not due to limitations in the method used or the device itself. However, the need for a significant number of exclusions demonstrate that we must continue the work to improve the probe housing used to overcome these issues in the future.

The participants included in the analysis were calm or asleep during the test, but the state of sleep was not assessed. It is known that sleep state affects the strength of HR response in older infants.^44^ Maternal factors such as advancing age and alcohol consumption have been identified as possible risk factors for neonatal encephalopathy,^45^ of which hypoxic–ischemic encephalopathy is a subgroup, and may influence the maturity of cerebral autoregulation and the tilt response. However, maternal factors were not registered in our study.

Accurate measurement of BP can be challenging in the neonate.^46^ We used an oscillometric technique since it is gentle and has low risk, although it has been reported to deviate from invasive intra-arterial BP.^47^ Invasive measurement of BP would be preferable but is ethically problematic to introduce in studies on healthy neonates.

The large diversity of responses makes it difficult to draw general conclusions. That some neonates presented with different responses at the 2 days is in line with earlier findings.^19^ The frequency of occurrence of some responses was found increased with increasing gestational age, but the neonates kept showing different responses. As all our neonates were born at term and only tested once at two consecutive days, we were unable to discover trends in cluster transitions. It is possible that repeated testing would have increased the reproducibility of the tests. However, repeated testing could also introduce test–retest–effects.^14^

Despite these limitations, our results demonstrate that healthy neonates react to a tilt test in a variety of ways. Continuous real-time Doppler ultrasound can monitor several important parameters such as BFV, HR, and PI, and can be used to detect instant changes in these parameters. There is a strong need for more research on the prognostic and diagnostic value of ultrasound-derived perfusion indices.^48^ Tracking the changes in Doppler-derived parameters for prolonged periods of time and combining several parameters into single scores may be fruitful avenues of research.

The diversity of normal cerebral hemodynamics in neonates makes it important to deploy large data sets to devise sensitive and specific algorithms for detecting abnormal and pathological patterns that may indicate immediate or prophylactic medical attention. Continuous real-time assessment of cerebral perfusion could potentially enable the clinician to adjust and individualize the management for a vulnerable patient. However, further research is warranted to study hemodynamics in relevant subgroups of patients such as premature babies during transition, asphyxia patients, and children with congenital heart disease during surgery.

In summary, we found that the NeoDoppler ultrasound system was able to monitor cerebral blood flow successfully during tilt test in 82% of the neonates and detect instant changes in cerebral hemodynamic parameters such as mean BFV, HR, and PI following tilt. Cluster analysis revealed five initial responses that were associated with specific subsequent development in cerebral mean BFV, HR, and PI the next 2 min. The clusters seemed not to be associated with specific changes in MAP. There is a diversity of normal responses to tilt in neonates, and further research is needed to assess their clinical relevance. It seems likely that the responses arise as an interaction between inherent features of the neonate and the specific experimental situation. The normal patterns are important as a point of reference for future ultrasound monitoring of sick children.

We believe that continuous real-time assessment of cerebral BFV can contribute to improved understanding of normal and pathological responses to circulatory stress.

## Supplementary information

Supplemental Figures.
