# Supplementary material for: Effects of tilt on cerebral hemodynamics measured by NeoDoppler in healthy neonates
Source: Pediatr Res. 2021 Jan 27;90(4):888–95. doi: 10.1038/s41390-020-01354-w (PMC8566239; doi:10.1038/s41390-020-01354-w)
Supplement: Supplementary file 1 — Supplemental Figures. [file 41390_2020_1354_MOESM1_ESM.pdf]

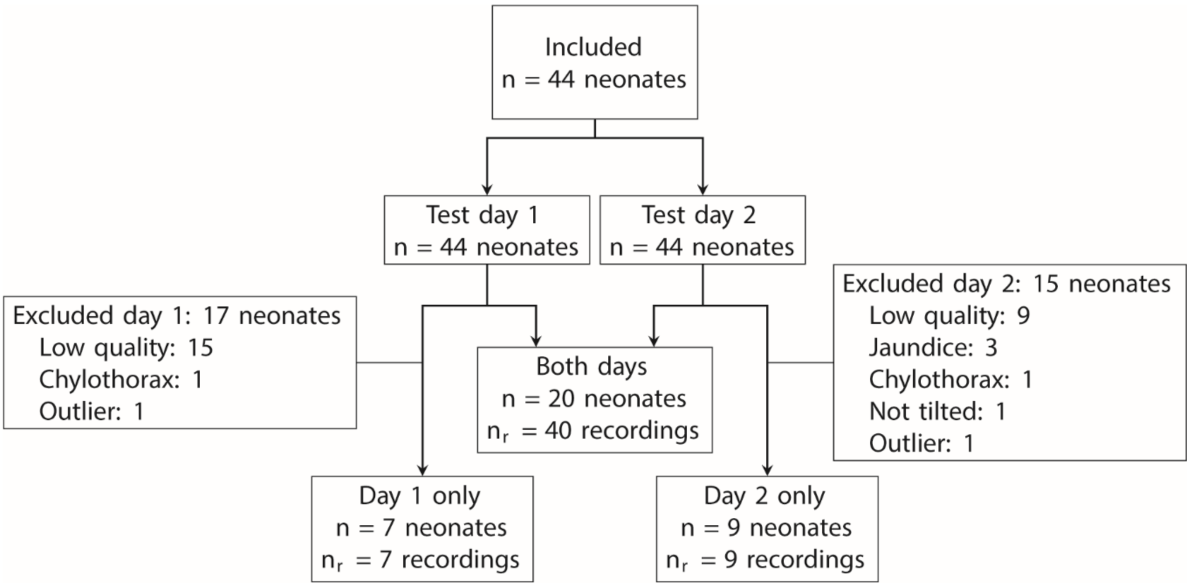

Supplemental Figure S1: Flow chart for included and excluded recordings. 44 neonates participated in a tilt test at the two first days after birth. After exclusions, 56 recordings from 36 different neonates were included for analysis. 20 neonates had recordings included from both tests.

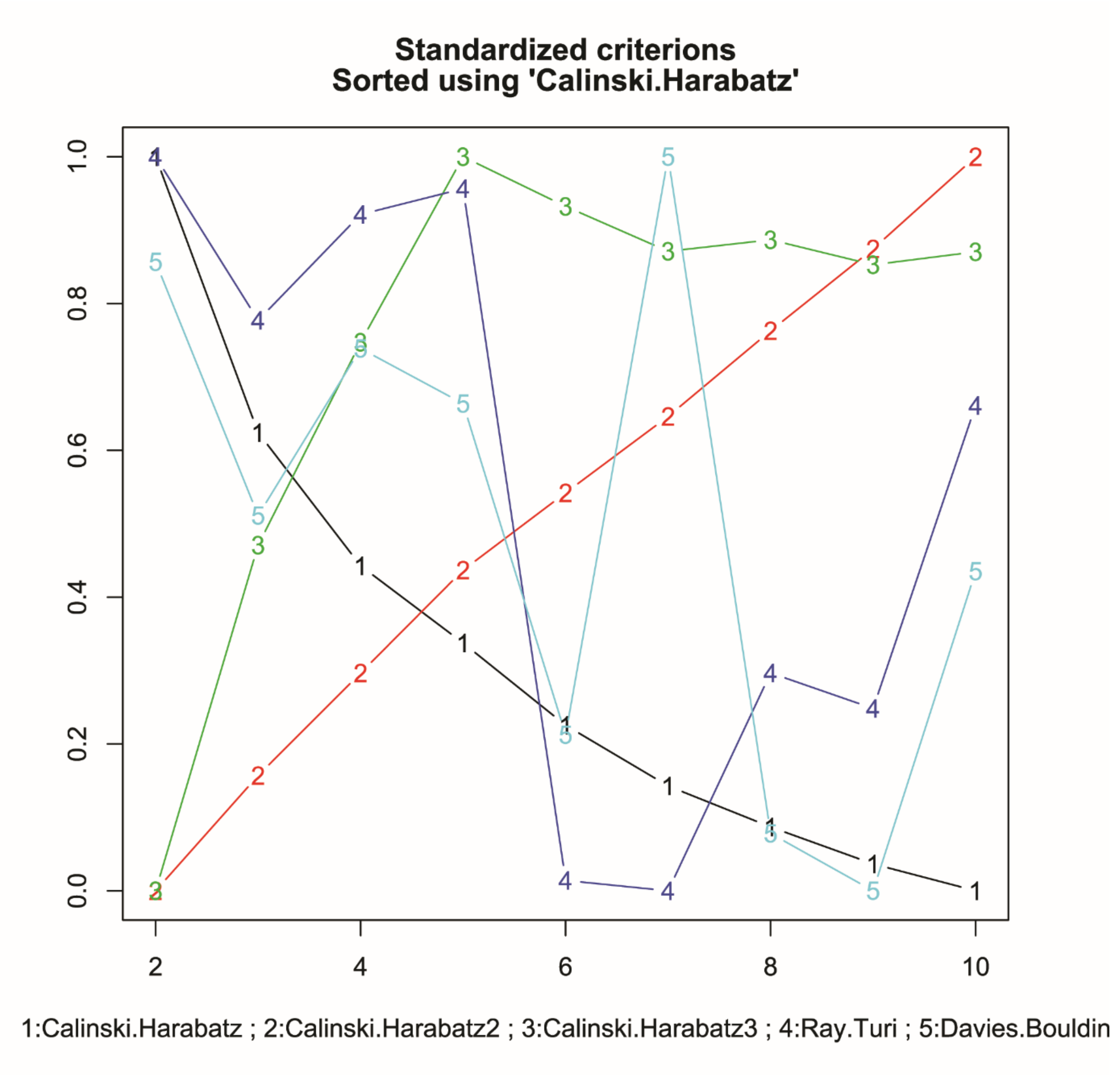

Supplemental Figure S2: Quality scores for deciding the optimal number of clusters. In k-means clustering, the optimal number of groups, *k*, is not known beforehand. The plot shows five different quality scores for increasing number of *k*. The scores are maximized by high between-cluster variation in combination with low within-cluster variation.
